# Chromatographic, Chemometric and Antioxidant Assessment of the Equivalence of Granules and Herbal Materials of Angelicae Sinensis Radix

**DOI:** 10.3390/medicines7060035

**Published:** 2020-06-23

**Authors:** Valentina Razmovski-Naumovski, Xian Zhou, Ho Yee Wong, Antony Kam, Jarryd Pearson, Kelvin Chan

**Affiliations:** 1 South Western Sydney Clinical School, School of Medicine, University of New South Wales, Sydney, NSW 2052, Australia; v.naumovski@unsw.edu.au; 2 NICM Health Research Institute, Western Sydney University, Penrith, NSW 2751, Australia; p.zhou@westernsydney.edu.au (X.Z.); j.pearson@westernsydney.edu.au (J.P.); 3 School of Pharmacy, The University of Sydney, A15, Science Rd, Camperdown, NSW 2006, Australia; ellewong613@gmail.com (H.Y.W.); k.antony@ntu.edu.sg (A.K.); 4 School of Biological Sciences, Nanyang Technological University, 60 Nanyang Drive, Singapore 63755, Singapore; 5 School of Pharmacy and Biomolecular Sciences, Liverpool John Moores University, Liverpool L3 3AF, UK

**Keywords:** Angelicae Sinensis Radix, antioxidant, Danggui, granules, herb, multivariate analysis, ultra-performance liquid chromatography

## Abstract

**Background:** Granules are a popular way of administrating herbal decoctions. However, there are no standardised quality control methods for granules, with few studies comparing the granules to traditional herbal decoctions. This study developed a multi-analytical platform to compare the quality of granule products to herb/decoction pieces of Angelicae Sinensis Radix (Danggui). **Methods:** A validated ultra-performance liquid chromatography coupled with photodiode array detector (UPLC-PDA) method quantitatively compared the aqueous extracts. Hierarchical agglomerative clustering analysis (HCA) and principal component analysis (PCA) clustered the samples according to three chemical compounds: ferulic acid, caffeic acid and Z-ligustilide. Ferric ion-reducing antioxidant power (FRAP) and 2,2-Diphenyl-1-picrylhydrazyl radical scavenging capacity (DPPH) assessed the antioxidant activity of the samples. **Results:** HCA and PCA allocated the samples into two main groups: granule products and herb/decoction pieces. Greater differentiation between the samples was obtained with three chemical markers compared to using one marker. The herb/decoction pieces group showed comparatively higher extraction yields and significantly higher DPPH and FRAP (*p* < 0.05), which was positively correlated to caffeic acid and ferulic acid, respectively. **Conclusions:** The results confirm the need for the quality assessment of granule products using more than one chemical marker for widespread practitioner and consumer use.

## 1. Introduction

Granule formulations have become the most popular delivery form for Chinese medicinal herbs and are used as an alternative to herb and decoction pieces in herbal prescriptions worldwide including China, Japan, USA and Europe [1]. For practitioners and consumers, granules are convenient in terms of easier administration (granules are added to water instead of boiling herbs in water which are then strained), transport (less bulky than herbs) and storage (protected from microbes and moisture). There is potential for better quality control of granules using good manufacturing practice (GMP) processes which would assure the reproducibility of products. This would promote clinical consistency as solvent ratios to herbs and boiling times of herbs/decoction pieces are not patient-dependent [2,3]. However, standardised quality control procedures for granules are limited. In recent times, ultra-performance liquid chromatography (UPLC) has analysed the granule formulations of popular herbs such as *Panax ginseng* (Araliaceae), *Salvia miltiorrhiza* (Lamiaceae), *Panax notoginseng* (Araliaceae) and other common composite formulae [2,4,5,6,7]. However, there are few comparative studies regarding the actual quality and efficacy of granules compared to the traditional herbal decoction, and the variations between granule formulations from different manufacturers [2,3,4]. This calls for a simple and rapid multi-method approach to guarantee the reliability and bioequivalence of herbal products to ensure their clinical efficacy [8].

In this study, the herb/decoction pieces and granule products of Angelicae Sinensis Radix, also known as Danggui in Chinese, are evaluated [9]. Danggui, the dried root of *Angelica* (A.) *sinensis* (Oliv.) Diels (Umbelliferae), is one of the most popular Chinese materia medica and is used in dietary supplements and cosmetics globally [10]. Originally listed as top grade in the Shennong’s Classic of Herbology and nowadays described as ‘female ginseng’, Danggui is used in gynaecological disorders such as painful dysmenorrhea, postpartum weakness and treating menopause [11]. The herb is known to regulate blood circulation, have antioxidant activity, and is widely used in cardiovascular diseases such as atherosclerosis and hypertension [12]. Danggui is present in over 80 composite formulae of traditional Chinese medicine (TCM).

Despite the popularity of Danggui, there is no quality assessment of Danggui granules [13]. With the general consensus of using a multi-method approach in assessing the quality of herbal products, the present study evaluated the differences between the Danggui samples using chromatography, chemometrics and antioxidant activity. Three chemical markers (ferulic acid, caffeic acid and Z-ligustilide) were quantified using the developed UPLC method. Hierarchical agglomerative clustering analysis (HCA) and principal component analysis (PCA) grouped the samples according to the content of the three markers. The results were compared to using either ferulic acid or Z-ligustilide as the single chemical marker as specified by the Pharmacopoeia of the People’s Republic of China (PPRC) [9] and World Health Organisation (WHO) guidelines, respectively [14]. Coupled with the statistical clustering analysis, correlating the chemical markers to antioxidant activity provided a comprehensive study of the differences between the products. Any variations between the products may imply possible pharmacological differences which need to be addressed in terms of correct dosages to patients.

## 2. Materials and Methods

### 2.1. Plant Materials and Reagents

Ten commercial Danggui granule products (coded as G1–G10) were produced by companies in mainland China, Hong Kong and Taiwan, and were either purchased from their distributors in Australia or directly from the manufacturers. Product names have been omitted as consent for disclosure was not sort. One herb (coded as R2) and four decoction piece samples (coded as R1, R3, R4, R5) were sourced from Min Xian, Gansu Province in China [9]. The region where the herbal material was sourced from is well known for Danggui and considered the best quality according to TCM. They were purchased from Australia, mainland China and Hong Kong. The samples were authenticated by Dr George Li from the Faculty of Pharmacy, The University of Sydney, Australia. The taxonomic identification was carried out macroscopically and microscopically according to the descriptions in the Pharmacopoeia of People’s Republic of China (PPRC) [9]. Voucher specimens were deposited at NICM Health Research Institute, Western Sydney University, Australia. They were labelled as for granules: G(number)(company)(date)AS and raw materials: R(number)(date)AS.

The three reference chemical markers (caffeic acid, ferulic acid and Z-ligustilide) were purchased from Chengdu Biopurify Phytochemicals Ltd. (Sichuan, China) and were graded > 98% HPLC purity. The compounds were verified with liquid chromatography–mass spectrometry (LC–MS). Chloroform, formic acid and acetonitrile were obtained from Ajax Finechem (Taren Point, Australia). Methanol was purchased from Fisher Scientific (Loughborough, UK) and ethyl acetate was purchased from Biolab Ltd. (Scoresby, Australia). Water was obtained from a Milli-Q Reagent Water System (Millipore, Burlington, MA, USA). All the solvents mentioned were HPLC-grade. For the antioxidant assays, DPPH, Trolox, sodium acetate trihydrate, glacial acetic acid, TPTZ (2, 4, 6-tripyridyl-s-triazine), hydrochloric (HCl) acid and ferric chloride hexahydrate were purchased from Sigma-Aldrich Corp (St. Louis, MO, USA).

### 2.2. Preparation of the Extracts and Standards

In this study, it was anticipated that the granule manufacturing process involved the large-scale extraction of herbs with boiling water to reflect the traditional decoction, followed by spray-drying or fluidised bed drying and formulation with excipients [5]. Thus, to remove most of the water-soluble excipients, 1 g of the Danggui granule sample was suspended in methanol (10 mL) and sonicated for 30 min at 40 °C. The sonicated mixture was centrifuged at 4000 rpm for 10 min and the supernatant removed. The extraction was repeated two more times. The combined supernatants were concentrated by a rotary evaporator to dryness at 50 °C. Here, the residue obtained from the granules after the methanol extraction was assumed to be equivalent to the raw herb water extract without excipients.

The herb and decoction pieces of Danggui were ground by an electric blender and passed through a 500 µm aperture sieve. The powder (1 g) was refluxed with boiling water (30 mL) for 30 min and the extraction repeated two more times. The sample was then centrifuged at 4000 rpm for 10 min. The supernatant was transferred and evaporated to dryness at 50 °C. This was followed by the same extraction procedure as described for the granule samples to allow comparison of the samples as methanol extracts. The solutions were prepared by re-dissolving the dry extract residues with methanol followed by filtrating into the final testing samples through the filter syringes (0.2 µm).

The individual standard stock solutions of the chemical markers caffeic acid, ferulic acid and Z-ligustilide were prepared at the concentration of 2 mg/mL in methanol. To minimise the impact of the stability, the standards and samples were freshly prepared each day and protected from heat, moisture and light.

### 2.3. Determination of Chemical Marker Content

UPLC analyses were performed using a Waters Acquity ultra performance liquid chromatography (UPLC)^®^ H series consisting of a H class quaternary solvent manager, an Acquity sample manager-FTN, an Acquity column oven and an Acquity Photodiode Array Detector (PDA) detector. The chromatographic separation was achieved using an Acquity UPLC BEH C18 column (50 mm × 2.1 mm, 1.7 µm) maintained at 40 °C [15].

The UPLC condition was based on our in-house HPLC method with modifications to the gradient condition [16,17,18]. The mobile phase consisted of 1% formic acid in water (A) and acetonitrile (B) (95:5, *v*/*v*), with a gradient elution as follows: 0–10 min, 5–12% B; 10–15 min, 12–20% B; 15–20 min, 20–100% B, 100% B for 5 min and reconditioning the column isocratically with 5% B for 4.5 min. The flow rate was 0.3 mL/min. The injection volume was 2 µL and the detection wavelength was set at 325 nm, which was similar to previous studies which monitored for ferulic acid and Z-ligustilide [15,17,19].

The UPLC method was validated in terms of linearity, repeatability and accuracy according to ICH guidelines [20]. Linearity testing was carried out by running six different concentrations of each chemical marker (caffeic acid (0.005–2 mg/mL), ferulic acid (0.005–2 mg/mL) and Z-ligustilide (0.01–0.3 mg/mL) in triplicate. Partial least square regression method was used to obtain the regression equations in the form of y = ax + b, where x is the concentration of the reference chemical marker and y was the peak area [21]. The limit of detection (LOD) and the limit of quantification (LOQ) were determined by standard deviation (SD) approach, where LOD = 3.33 × (SD of y-intercept/mean of slope) and LOQ = 10 × (SD of y-intercept/mean of slope). For repeatability, the intra-day precision was evaluated by running six concentrations of each marker three times within a day, whilst inter-day precision was examined on three separate consecutive days. To determine the accuracy of the method, a recovery assay was performed in triplicate by spiking two known concentrations (100 and 150 µg/mL) of the mixed standards (caffeic acid, ferulic acid) to one representative decoction piece and granule sample [22]. Percentage recovery (%) ± RSD was calculated by the equation: % = ((mean detected content − mean original content)/mean of spike content) × 100%.

### 2.4. Antioxidant Activity Assays

The 2,2-diphenyl-1-picrylhydrazyl (DPPH) assay was performed as previously described [23]. The test samples were mixed with DPPH radical solution (0.24 mg/mL DPPH in methanol) and incubated for 30 min in the dark. The absorbance was determined at 515 nm. (±)-6-Hydroxy-2,5,7,8-tetramethylchromane-2-carboxylic acid (Trolox) was used for the calibration curve. All values were expressed as milligrams Trolox equivalents (TE) per gram of dried weight (DW) (mg TE/g DW).

The ferric ion reducing antioxidant power (FRAP) assay was performed as previously described [24]. The FRAP working solution was prepared by mixing 10 volumes of 300 mM acetate buffer (pH 3.6), 1 volume of 10 mM 2,4,6-tris(2-pyridyl)-s-triazine (TPTZ) in 40 mM HCl and 1 volume of 20 mM ferric chloride (FeCl_3_·6H_2_O). The test samples were mixed with pre-warmed FRAP reagent (37 °C) and incubated for 30 min at 37 °C. The absorbance was measured at 595 nm. The standard curve and the results of TE were obtained by the same approach as described above.

### 2.5. Statistical Analyses

The yields (reported as percentage of the dry weight of the herb) is the mean of three extractions. One of the extracts from the same sample was analysed three times by UPLC, with the final quantitative results from UPLC analyses expressed as the mean ± standard deviation (SD). Quantitative results were reported as milligrams per grams of the DW of the raw herb (mg/g) equivalent. Non-parametric test (SPSS 20.0 software, IBM, Chicago, IL, USA) was conducted to determine whether the content of each chemical marker analysed by UPLC was significantly different between individual samples.

The chosen markers were considered as variables in the following HCA and PCA statistical analysis. HCA grouped the individual Danggui samples into clusters based on the degree of the similarity of the variables. The HCA results were expressed as a dendrogram using Ward’s linkage algorithm and squared Euclidean distances (SPSS 20.0 software, IBM, Chicago, IL, USA). The different linkage criteria applied in the dendrogram revealed the degree of similarity between each sample. The length of the linkage between each sample/group represents the degree of similarity. Thus, the shorter the linkage, the more similarity there is between each group. PCA was performed using XLSTAT 2019.1 by Addinsoft (New York, USA) which reduced the original variables into two major principal components (PCs). These two PCs maintained the greatest possible variance of the original variables (three chemical markers) [7]. The PCA results were represented in a biplot (score plot and loading plot), where the score plot showed the clusters and outliers of the samples, and the loading plot demonstrated the correlation of the PC to the original variables. In the biplot, a point represented each individual sample, and the distance allocated between samples revealed the degree of their similarity in terms of the content of the chemical markers.

For the antioxidant assays, the data were expressed as the mean ± standard deviation (SD) of three repeat measurements and was analysed using independent-samples t-test and non-parametric analysis by SPSS. For this study, *p* < 0.05 was considered as statistically significant. Pearson correlation coefficient (r) by SPSS (20.0 software, IBM, Chicago, IL, USA) evaluated the strength of the correlation of the chemical markers to the antioxidant activities.

## 3. Results

### 3.1. Extraction Yields

The mean yield of each sample is shown in Table 1. The yield results of the granule products were converted according to their concentrated ratio (as listed on the package) so that comparison to the original herbal material could be made. The yields of the herb/decoction pieces (33.2–44.8%) as a group were comparatively higher than that of the granules (2.7–12.9%).

### 3.2. UPLC-PDA Quantification of the Chemical Markers

**Figure 1:**
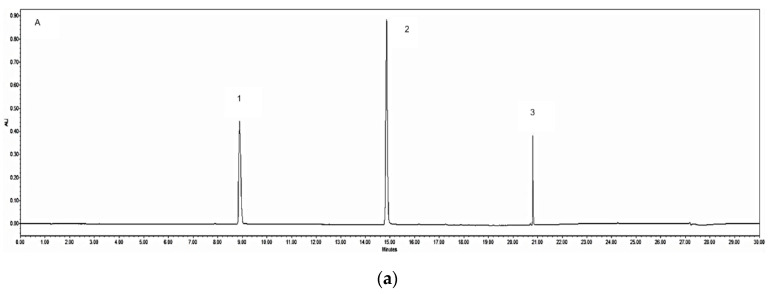

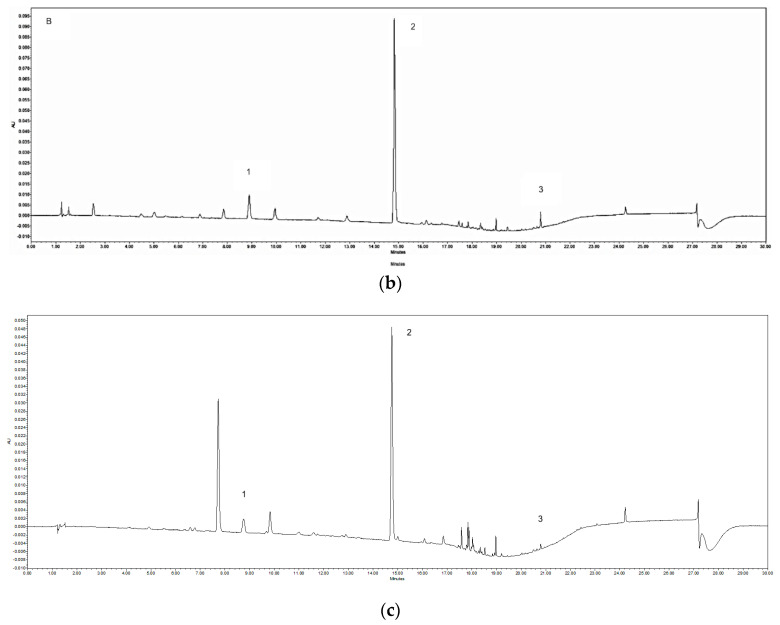
UPLC chromatograms detected under the developed mobile phase system at 325 nm. These chromatograms show: (**a**) three marker compounds: 1 = caffeic acid (0.1 mg/mL), 2 = ferulic acid (0.2 mg/mL), 3 = Z-ligustilide (0.1 mg/mL); (**b**) granule 1 (G1) sample (10 mg/mL); (**c**) raw herb 2 (R2) sample (10 mg/mL). AU = absorbance units.

The representative chromatograms of the mixed chemical markers, the granule (G1) and herb (R2) extracts are shown in Figure 1, with a total run time of 30 min. In this study, the three chemical markers, caffeic acid, ferulic acid and Z-ligustilide and their calibration curves produced good correlations between the peak area and concentration as shown in Table 2, with the correlation coefficients r^2^ > 0.997 for all analytes. The LODs and LOQs were in the range of 0.701–3.268 μg/mL and 2.106–9.813 μg/mL, respectively. The intra-day and inter-day RSD were 1.5–2.77% and 2.6–4.11%, respectively (Table 2). This suggests that the method had reasonable instrumental and method precision [22].

The addition of known amounts of the compounds to the samples is recommended for recovery testing of herbal compounds [22]. Granule sample 2 (G2) and decoction piece sample 3 (R3) were randomly chosen as representative Danggui samples from each group. The average recoveries (%) were for G2: 89.3 ± 1.1% (caffeic acid) and 99.7 ± 1.2% (ferulic acid); R3: 94.1 ± 2.1% (caffeic acid) and 99.6 ± 2.1% (ferulic acid).

The developed UPLC-PDA method simultaneously quantified the three marker compounds in the Danggui water extract herb and granule samples, and the results are shown in Table 1. In this study, the amount of caffeic acid, ferulic acid and Z-ligustilide in all the samples ranged from 0.004–0.041, 0.030–0.503 and 0.005–0.526 mg/g DW, respectively. In the granule samples, G5 and G10 had a relatively higher amount of Z-ligustilide (0.526 mg/g DW and 0.183 mg/g DW, respectively) compared to the rest of the samples.

Nonparametric independent-samples t-testing of the raw herb samples revealed that R2 had significantly higher ferulic acid (*p* < 0.05) and caffeic acid (*p* < 0.05), and there was no significant difference in Z-ligustilide content (*p* > 0.05).

Both caffeic acid and Z-ligustilide were not significantly different between the granule and decoction piece/raw herb groups (*p* > 0.05). However, the amount of ferulic acid was found to be significantly different (*p* < 0.05) between the two groups. In terms of ferulic acid and caffeic acid, G7 (higher content) and G10 were significantly different (*p* < 0.05). In terms of Z-ligustilide, G5 (higher content) (*p* < 0.05) and G10 (*p* < 0.05) were significantly different.

### 3.3. Multivariate Analysis Using HCA and PCA

**Figure 2:**
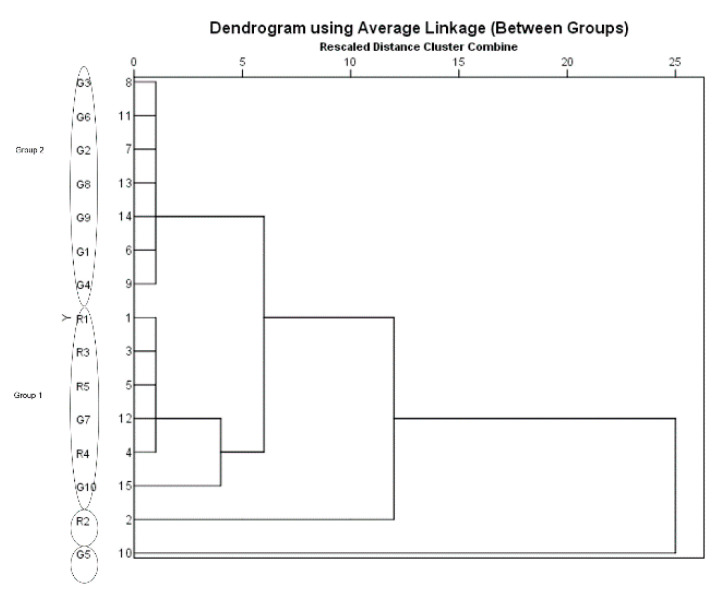
Hierarchical agglomerative clustering analysis (HCA) dendrogram of Danggui samples using SPSS 20.0 software (Chicago, USA). Ward’s method as amalgamation rule and the squared Euclidean distance as metric were employed to set up the clusters. The length of the linkage between each sample/group represents the degree of similarity. G: granule samples; R: raw herbs/decoction piece samples. Group 1: R1, R3, R5, G7, R4, G10; Group 2: G1, G2, G3, G4, G6, G8, G9. R2 and G5 are outliers.

According to the dendrogram generated from HCA, the majority of the samples were divided into two main clusters. Specifically, R1, R3, R4, R5, G7 and G10 (relatively higher amounts of caffeic acid and ferulic acid) were classified into one cluster (Group 1), whereas G1–G4, G6, G8 and G9 were grouped into another cluster (Group 2) representing relatively lower amount of the marker acids. G5 (highest amount of Z-ligustilide) and R2 (highest amount of ferulic acid) were different to these two main groups (Figure 2).

**Figure 3:**
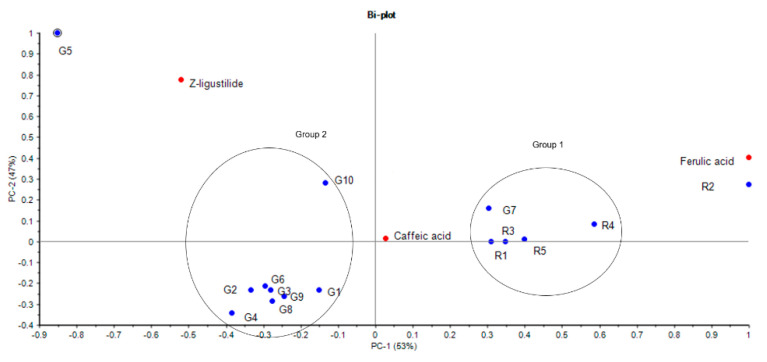
Biplot from principal component analysis (PCA) of Danggui samples (PC1 vs. PC2) based on the three components Z-ligustilide, caffeic acid and ferulic acid using Unscrambler 10.3 from Camo AS software (Trondheim, Norway). In the biplot, a point represented each individual sample, and the distance allocated between samples, revealed the degree of their similarity in terms of the content of the chemical markers. PC: principal component. G: granule samples; R: raw herbs/decoction piece samples. Group 1: R1, R3, R5, G7, R4; Group 2: G1, G2, G3, G4, G6, G8, G9.

PCA was also performed to determine the main chemical markers influencing the equivalence of Danggui raw materials and granules. Based on eigenvalues > 1, the first two principal components (PC), PC1 and PC2, were used to differentiate the samples according to the input data. From the result, the first two PCs could explain 53% and 47% of the variance of the three chemical markers, respectively. According to the loading matrices from the PCA biplot, the test samples were separated in PC1 by the differences in the chemical content of ferulic acid and caffeic acid, whilst PC2 was mainly due to the chemical content of Z-ligustilide. Similar to the results of hierarchical clustering, two major groups are set up in the PCA biplot (Figure 3). The decoction pieces and G7 (Group 1) were in close proximity and showed a higher content of caffeic acid and ferulic acid, with R2 demonstrating the highest amount of caffeic acid and ferulic acid. G5 was considered as an outlier of the samples due to its excessively high amount of Z-ligustilide. The PCA loading plot indicates that the Z-ligustilide content may have more influence on the discrimination of G5 and G10. The rest of the granules (Group 2) were near each other and represented generally lower amounts of the three chemical markers.

The HCA plot of the three markers was compared to using a single marker and the combination of the markers (Supplementary Material). Ferulic acid as the sole marker grouped G5 with the granules, showing no distinct difference (Figure S1). The HCA of the two markers (ferulic acid and Z-ligustilide) showed similar results to the original three marker HCA, with R2 showing more similarity to the decoction pieces (Figure S2). Using Z-ligustilide, all samples were grouped together, with G5 on its own (Figure S3). Using caffeic acid, there were three groups: G7 and G10 grouped with R2; G4 and G5 was with the rest of the herb/decoction pieces, with G5 showing some similarity with the granules (Figure S4).

### 3.4. Antioxidant Activity

In the DPPH and FRAP assays (Table 3), all the samples showed antioxidant activity, with R2 (highest amount of ferulic acid) showing significantly higher activity in both assays using independent-samples t-test (*p* = 0.017 and 0.002, respectively), whereas G5 (highest amount of z-ligustilide) was comparable (*p* = 0.421 and 0.483, respectively). A significant difference in antioxidant activity was shown between the herb/decoction piece samples as a group and the granules as a group (*p* < 0.05) in the FRAP assay. For the two major groups established by HCA and PCA, DPPH and FRAP antioxidant activities were compared by independent-samples t-test and found a significant difference (*p* = 0.027) between Group 1 and 2 in the FRAP assay.

Pearson correlation coefficient analysis investigated the correlation between antioxidant activity and the chemical markers of all the Danggui samples (Table 4). Positive and significant correlations were observed between the amount of ferulic acid and the antioxidant activities of the FRAP assay (0.791, *p* < 0.01). Caffeic acid showed significant correlation with the antioxidant activities of Danggui as measured by the DPPH assay (0.582, *p* < 0.05). In contrast, the amount of Z-ligustilide and samples’ antioxidant activity was negatively and not significantly correlated to either DPPH and FRAP assay (−0.202 and −0.229, *p* > 0.05).

## 4. Discussion

As demand grows for traditional Chinese medicines, so does the need for efficient ways of administrating herbal medicines. Thus, it is important to compare new formulations such as granules to the original herb. This is the first study that compares Danggui granules to the raw products. The yield (reported as percentage of the dry weight of the herb) is indicative of the herb dosage a patient is consuming. The yields were higher for the herb/decoction pieces and were lower than the 48% water-soluble extractives in the Hong Kong Chinese Materia Medica Standards (HKCMMS) [25]. In comparison, the yield of the granule products was lower. Granule size was nonuniform in the samples, and this will affect the extraction process above. Smaller particles may be extracted more efficiently or be missed as they make their way to the bottom of the bottle. To minimise this variability, each granule bottle was shaken before sampling to redistribute the particles [26,27].

The quality control of traditional Chinese medicines and their products is a challenge for industry due to the complexity of the formulations (using a holistic approach to treat disease), as well as high outlay costs for analytical instrumentation. Danggui has a complex chromatogram because of the number of individual constituents, the possible degradation and isomerisation of the organic acids and phthalides present [28,29]. Qualitative approaches such as thin layer chromatography (TLC) are highly recommended by pharmacopoeias and monographs to compare fingerprints of samples; however, it is does not usually allow for the quantification of compounds which will confirm their quality [25,30]. For this quality study on Danggui, the visual analysis of the TLC result failed to accurately determine the quantitative difference between the compounds of the samples as the LOD and concentrations of the compounds were low and close to signal noise, and calibration curves could not be constructed (data not shown).

Other studies have used UPLC coupled with MS to investigate the chemical profile of *A. sinensis* [15,31]. This analysis would have expensive set up costs for examining herbal material. To separate the polar and non-polar constituents within a reasonably short running time, a UPLC condition was determined and optimised in this study. By adding 1% formic acid to water, the solvent system showed good separation of the constituents simultaneously, with a run time of 30 min (compared to 60 min for high-performance liquid chromatography methods) and good separation [32]. The optimised UPLC method and resulting chromatograms were able to quantify caffeic acid, ferulic acid and Z-ligustilide.

The disparity of the ferulic acid, caffeic acid and Z-ligustilide content between the granule samples indicates differences in the manufacturing processing of Danggui which may not mimic traditional water decoctions of TCM. It is interesting to note that R2 was a raw herb sample rather than a decoction piece (which has gone through a processing procedure such as smoke-drying). Its extraction in water favoured the polar compounds such as the organic acids. However, it has been reported that techniques such as steam distillation and other solvents such as ethanol may be used to enhance the extraction of the less polar components in the herb for granule production at the expense of the polar acids such as ferulic acid. Spray or vacuum drying may be used for heat sensitive compounds and for compounds in trace amounts [33]. For water insoluble compounds such as Z-ligustilide, one company mentions the use of carbon dioxide extraction [34]. Studies have established pharmaceutical approaches using methanol and hexane extraction to obtain a high content of Z-ligustilide as a lead compound for pharmacological studies [17,28,35]. Another issue could be adulteration, in which the extracts may be spiked with the known marker compound to reach the regulatory amount [14].

As differences in the chemical content of Danggui products could affect their efficacy, the identification and quantification of chemical markers is necessary to determine the quality of Danggui granules. In the study, the amount of ferulic acid in the samples (0.003–0.05%) was less than the 0.05% minimum requirement as stated in the PPRC for the quality assessment of Danggui [9]. In the PPRC, 70% ethanol is the nominated solvent, with no less than 45% ethanol-soluble extractives. This solvent will give a different chemical profile compared to water as a solvent which is used in home decoctions. However, no information is given in the PPRC regarding the standard amount of caffeic acid and Z-ligustilide for Danggui. The monograph for Radix Angelicae Sinensis states that a “sample contains not less than 0.6% (of Z-ligustilide) calculated with reference to the dried substance” [25]. In this study, Z-ligustilide in most of the samples were lower than the monograph and the standard range of 0.5–5% as reported by the WHO which is based on 100% methanol as the solvent [14,28]. In addition, the amount of Z-ligustilide detected in the extracts (0.005–0.526 mg/g DW) was lower due to the extraction in water (traditional decoction) than the content (1.26–37.7 mg/g from non-water solvents) found in previous studies [17,29,36].

HCA and PCA differentiated the Danggui samples based on the contents of caffeic acid, ferulic acid and Z-ligustilide on their own and in combination. In this study, a minimum of two chemical marker compounds (ferulic acid and Z-ligustilide) was required to differentiate Danggui products. This agrees with our previous findings where five rather than the nominated three chemical markers were required to differentiate raw and granule products of *Panax notoginseng* [7]. Thus, it is recommended that the WHO, the PPRC and other pharmacopoeias/monographs incorporate a minimum percentage value of at least two chemical standards, which will reflect traditional water extracts.

DPPH, along with FRAP, are commonly used to measure antioxidant activity and the methods with other herbal products have been widely published. Unlike biological cell assays, these assays have stable reaction responses and are cheap and quick for industry use. The contribution of caffeic acid and ferulic acid to antioxidant activity of Danggui was confirmed in a previous study [37]. Our findings indicated that phenolic acids such as ferulic acid are the key determinants influencing the antioxidant activities of Danggui as found in a previous study [38]. Thus, the chemical content of ferulic acid is an important chemical marker to ensure the correlation of the antioxidant activities to the different Danggui samples. One study revealed that Danggui extracts prepared with either water or 20% ethanol with an extraction time of 15 min yielded the best antioxidant activity [39]. As expected, Z-ligustilide did not correlate to antioxidant activity.

A limitation of the present study is that the Danggui was sourced from one region which is the region recommended for quality Danggui. As expected, the results showed that the decoction pieces were consistent in composition. Future studies could include comparing granules to raw decoctions in clinical trials to gauge clinical efficacy.

## 5. Conclusions

In the present study, UPLC coupled with multivariate analysis and antioxidant activity provided a rapid method for assessing differences in Danggui products. Comprehensive quality standardisation processes in pharmacopoeias and monograph publications are required to guide the regulation and standardisation of the production of commercial herbal granules. With the increased use of herbal medicinal granules around the world, this study will provide important information for standardisation committees, industry, practitioners and consumers on the quality control of herbs and its medicinal products. It is vital that patients are better informed about their health and treatment choices and are aware of what they are consuming. More importantly, practitioners will need to determine the correct dosages for their patients so that they do not undermine the efficacy of the herb and the patient’s care. Thus, granule dosages would need to equate to the decocted raw product.

## Figures and Tables

**Table 1:** Average contents (mg/g, means ± SD, n = 3) of the chemical markers in the ten Danggui granule samples (G1–G10) and the five herb/decoction piece samples (R1–R5) analysed by UPLC-PDA.

Sample	Origin	Granule to Herb Ratio ^c^	Yield ^d^ % (with Ratio)	Caffeic Acid (mg/g)	Ferulic Acid (mg/g)	Z-Ligustilide (mg/g)
G1 ^a^	Guangxi	1:3	12.7 (4.2)	0.0153 ± 0.001	0.111 ± 0.008	0.0105 ± 0.001
G2 ^b^	Guangdong	1:5	31.7 (6.3)	0.0155 ± 0.000	0.0631 ± 0.002	0.0359 ± 0.001
G3 ^b^	China	1:5	28.5 (5.7)	0.0142 ± 0.000	0.0772 ± 0.002	0.0284 ± 0.001
G4 ^b^	Sichuan	1:10	27.3 (2.7)	0.00491 ± 0.000	0.0300 ± 0.001	0.00460 ± 0.000
G5 ^b^	Taichung	1:6	31.3 (5.2)	0.00598 ± 0.000	0.145 ± 0.008	0.526 ± 0.032 ^***^
G6 ^a^	Beijing	1:5	27.1 (5.4)	0.0179 ± 0.000	0.0764 ± 0.003	0.0367 ± 0.001
G7 ^a^	Jiangsu	2:5	32.3 (12.9)	0.0394 ± 0.002 ^**^	0.299 ± 0.014 ^**^	0.0810 ± 0.004
G8 ^a^	Guangdong	3:10	28.2 (8.5)	0.0122 ± 0.001	0.0688 ± 0.004	0.00923 ± 0.001
G9 ^a^	Sichuan	1:5	35.4 (7.1)	0.0113 ± 0.001	0.0808 ± 0.009	0.0129 ± 0.001
G10 ^a^	Guangdong	1:3.3	29.5 (8.9)	0.0312 ± 0.002 ^**^	0.206 ± 0.015 ^**^	0.183 ± 0.012 ^***^
R1			44.8	ND	0.274 ± 0.008	0.0262 ± 0.003
R2			42.1	0.0407 ± 0.008 ^*^	0.503 ± 0.074 ^*^	0.0245 ± 0.003
R3			42.3	ND	0.284 ± 0.013	0.0215 ± 0.003
R4			41.8	0.00455 ± 0.000	0.361 ± 0.006	0.0168 ± 0.001
R5			33.2	0.00396 ± 0.001	0.299 ± 0.013	0.0177 ± 0.002

G = granule; SD = standard deviation; ND = not detected. ^a^ Hospital-grade, use as directed by doctor, ^b^ Available in Australia; ^c^ Granule to raw herb ratio as specified by the manufacturer, thus 1 g granule is produced by 3 g herb etc.; ^d^ Average yield converted by granule ratio; ^*^ Significantly different within the raw samples (*p* < 0.05); ^**^ Significantly different within the granule samples (*p* < 0.05); ^***^ Significantly different within the granule samples (*p* < 0.05).

**Table 2:** Calibration curves, detection limits and quantification limits (*n* = 6) of the three chemical markers in Danggui by UPLC-PDA.

Compound	Regression Equation	R^2^	LOD (µg/mL)	LOQ (µg/mL)	Intra-Day RSD (%) (*n* = 6)	Inter-Day RSD (%) (*n* = 3)
Caffeic acid	y = 1.9561x + 0.0108	0.998	1.496	4.492	2.770	2.598
Ferulic acid	y = 1.9915x − 3.1394	0.999	0.701	2.106	1.496	2.790
Z-Ligustilide	y = 0.6409x − 0.0103	0.997	3.268	9.813	2.725	4.108

Relative standard deviation RSD (%) = 100 × standard deviation (SD)/mean; y, peak area; x, the concentration of each reference chemical marker (mg/mL); R^2^, coefficient of determination; LOD, limit of detection (3.33 × (SD of y-intercept/mean of slope)); LOQ, limit of quantification (10 × (SD of y-intercept/mean of slope)).

**Table 3:** Trolox equivalent (TE) of the granule and herb/decoction piece samples of dried weight (DW) using DPPH and FRAP assays, respectively.

	DPPH Assay ^a^	FRAP Assay ^a^
Sample	mg TE/g of DW ± SD	mg TE/g of DW ± SD
G1	1.79 ± 0.27	9.96 ± 4.90
G2	3.32 ± 0.29	13.60 ± 0.41
G3	2.83 ± 0.20	9.29 ± 0.27
G4	1.76 ± 0.04	6.21 ± 0.62
G5	2.29 ± 0.45	10.75 ± 0.30
G6	3.02 ± 0.44	10.28 ± 0.45
G7	6.33 ± 0.54	26.30 ± 0.36
G8	7.33 ± 0.32	30.82 ± 1.71
G9	4.02 ± 0.92	16.03 ± 0.05
G10	3.34 ± 0.66	13.09 ± 0.19
R1	3.26 ± 0.81	50.34 ± 10.21
R2	8.10 ± 0.59	69.38 ± 1.82
R3	3.05 ± 0.36	23.04 ± 5.21
R4	4.76 ± 0.42	25.73 ± 4.48
R5	2.76 ± 0.09	32.06 ± 4.07

^a^ Values were the average of triplicate tests; SD = standard deviation.

**Table 4:** Pearson correlation between the three chemical markers and antioxidant activities of the samples in the DPPH and FRAP assays.

	Assay	DPPH	FRAP
Markers			
Caffeic acid		0.582 *	0.257
Ferulic acid		0.507	0.791 **
Z-ligustilide		−0.202	−0.229

* Correlation is significant at the 0.05 level (two-tailed). ** Correlation is significant at the 0.01 level (two-tailed).

## Supplementary Materials

The following are available online at https://www.mdpi.com/2305-6320/7/6/35/s1, Hierarchical agglomerative clustering analysis (HCA) dendrograms of Danggui samples using SPSS 20.0 software (IBM, Chicago, IL, USA). Dendrograms show different combinations of the markers. Ward’s method as amalgamation rule and the squared Euclidean distance as metric were employed to set up the clusters. G: granule samples; R: raw herbs/decoction piece samples. Figure S1: Ferulic acid as the single marker, Figure S2: Ferulic acid and Z-ligustilide as the two markers, Figure S3: Z-ligustilide as the single marker, Figure S4: Caffeic acid as the single marker.
