# Supplementary material for: Chromatographic, Chemometric and Antioxidant Assessment of the Equivalence of Granules and Herbal Materials of Angelicae Sinensis Radix
Source: Medicines (Basel). 2020 Jun 23;7(6):35. doi: 10.3390/medicines7060035 (PMC7345935; doi:10.3390/medicines7060035)
Supplement: Supplementary file 1 [file medicines-07-00035-s001.pdf]

Supplementary Materials: Chromatographic, Chemometric and Antioxidant Assessment of the Equivalence of Granules and Herbal Materials of Angelicae Sinensis Radix

Valentina Razmovski-Naumovski, Xian Zhou, Ho Yee Wong, Antony Kam, Jarryd Pearson and Kelvin Chan

Supplementary Materials: Hierarchical agglomerative clustering analysis (HCA) dendrograms of Danggui samples using SPSS 20.0 software (Chicago, USA). Dendograms show different combinations of the markers. Ward's method as amalgamation rule and the squared Euclidean distance as metric were employed to set up the clusters. G: granule samples; R: raw herbs/decoction piece samples.

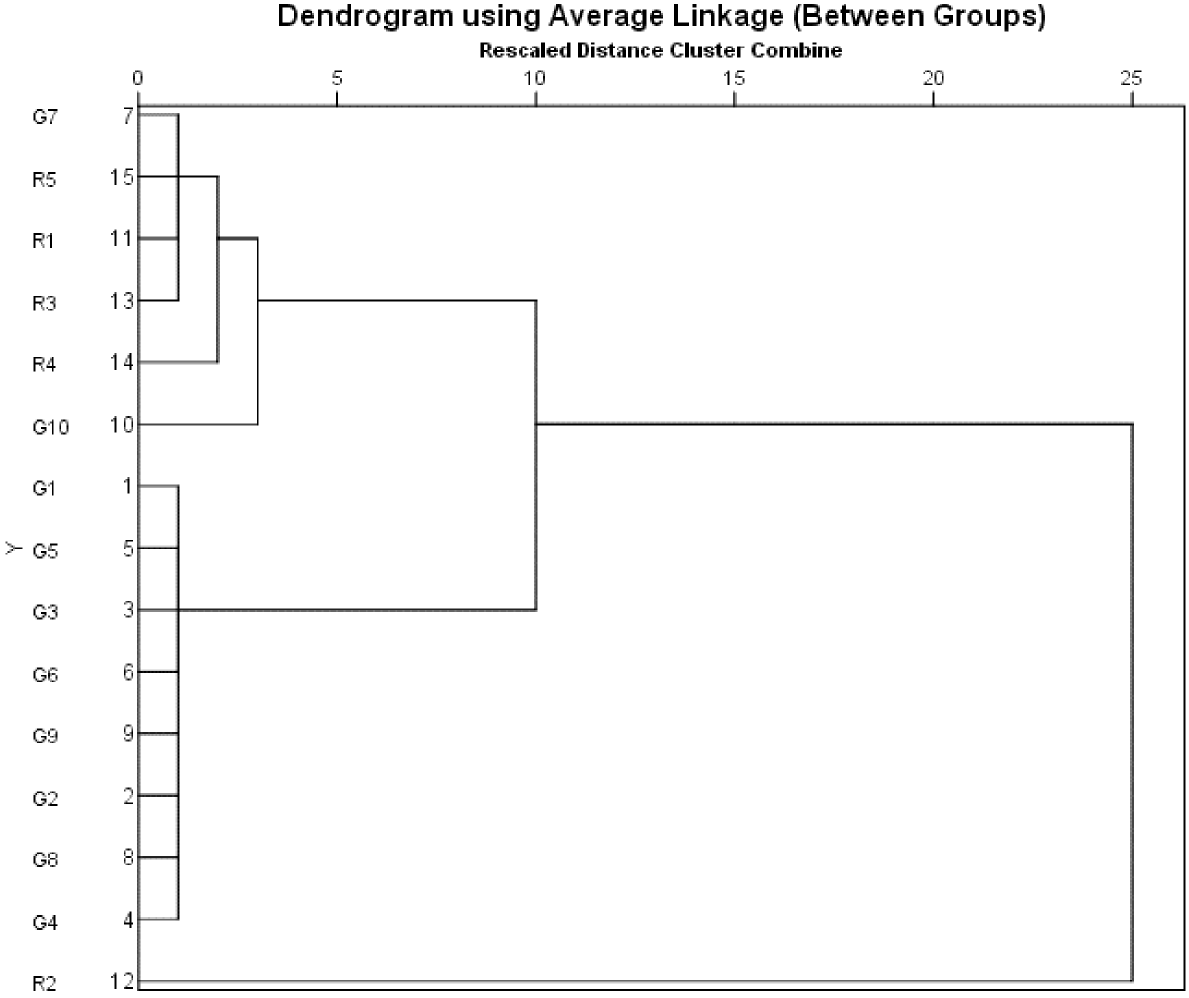

Figure S1. Ferulic acid as the single marker.

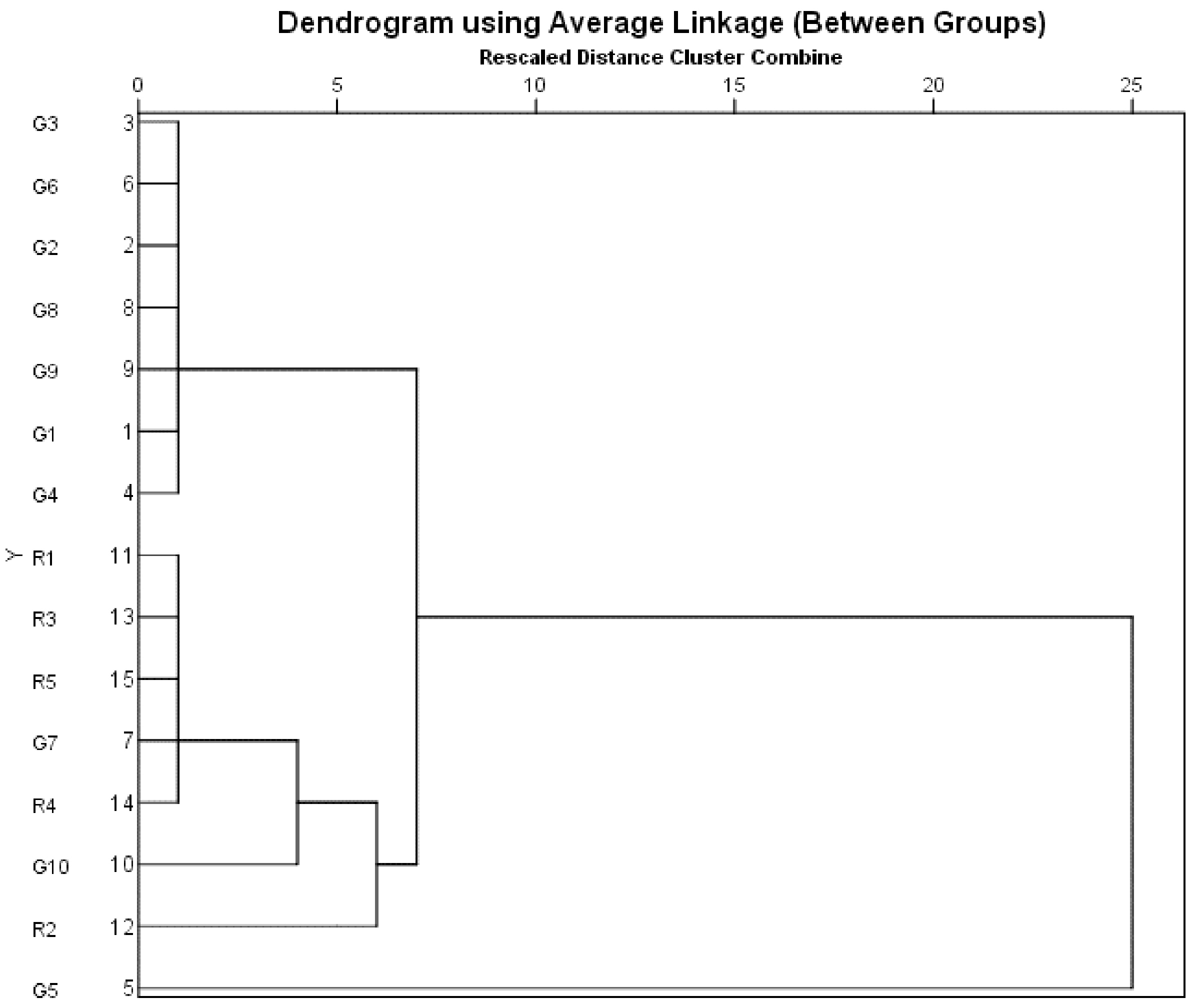

Figure S2. Ferulic acid and Z-ligustilide as the two markers.

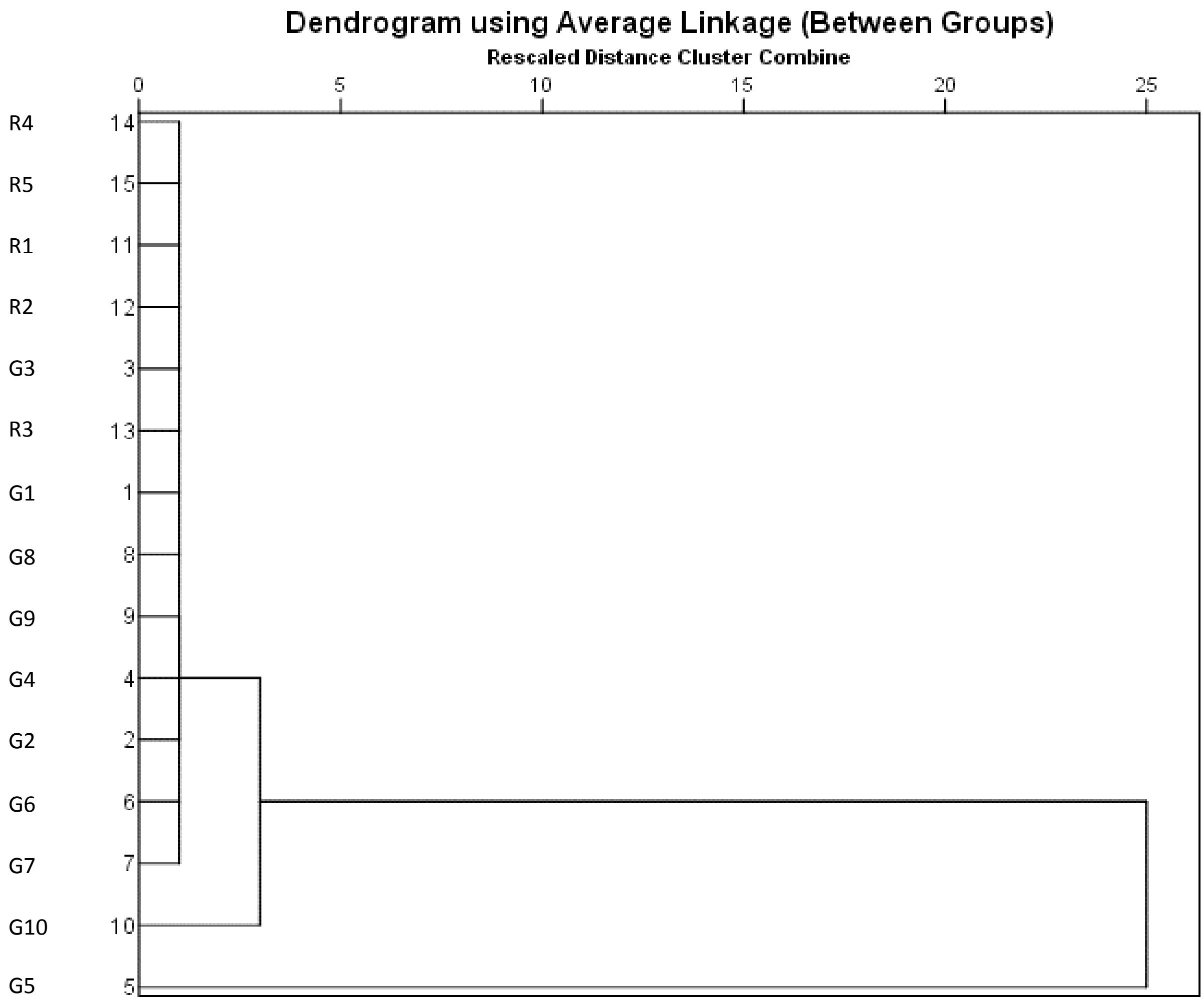

Figure S3. Z-ligustilide as the single marker.

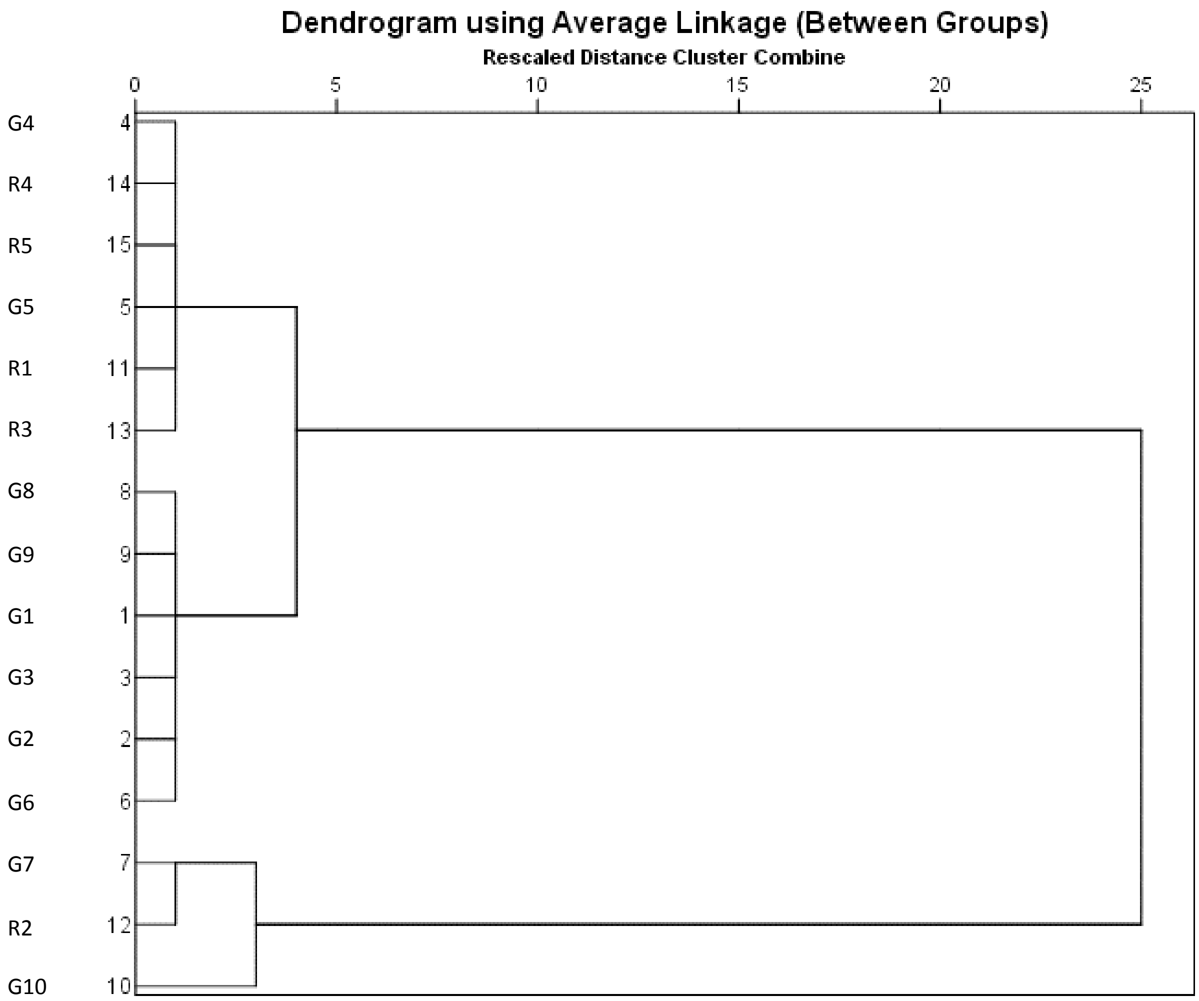

Figure S4. Caffeic acid as the single marker.
